# Genotoxicity assessment of 4-methylimidazole: regulatory perspectives

**DOI:** 10.1186/s41021-016-0050-z

**Published:** 2016-11-01

**Authors:** Takeshi Morita, Chikako Uneyama

**Affiliations:** 1 Division of Risk Assessment, National Institute of Health Sciences, 1-18-1 Kamiyoga, Setagaya-ku, Tokyo 158-8501 Japan; 2 Division of Safety Information on Drug and Food, National Institute of Health Sciences, 1-18-1 Kamiyoga, Setagaya-ku, Tokyo 158-8501 Japan

**Keywords:** 4-Methylimidazole, Caramel colors, Carcinogenicity, Genotoxicity, Risk assessment

## Abstract

4-Methylimidazole (4-MI) is formed as a result of the Maillard reaction process, and therefore is found in many foods and beverages. It is also found in soft drinks (i.e., cola) as a by-product in the production of some caramel colors. NTP bioassays revealed clear evidence of lung carcinogenicity of 4-MI in male and female mice, but not in rats and then IARC classified 4-MI as group 2B carcinogen. Genotoxicity studies with 4-MI were negative in the Ames tests and in the erythrocyte micronucleus tests with mice or rats. US California EPA (CEPA) evaluated the testing has not been adequately comprehensive to rule out a genotoxic mode of action; as target tissue of the carcinogenicity of 4-MI was lung, the lung should be used as a source tissue for in vitro metabolic activation system. Thus, CEPA defined the No Significant Risk Level (NSRL) for 10^−5^ lifetime risk level of cancer by 4-MI as 29 μg/day based on the non-threshold approach. As higher levels of 4-MI than the NSRL were identified in some kinds of cola, health concerns of 4-MI were drawn the attention. On the other hand, other regulatory bodies (e.g., European Food Safety Authority, EFSA) showed no concerns of 4-MI from the use of caramel colors in food. EFSA evaluated 4-MI is not genotoxic, so, non-observed adverse effect level of 4-MI was considered to be 80 mg/kg/day. In this paper, genotoxic assessments of 4-MI in different regulatory bodies are presented and the risk evaluation of 4-MI is discussed based on new genotoxicity data.

## Background

4-Methylimidazole (4-MI) will be present or subject to unavoidable formation during cooking in certain foods and beverages, including coffee, carbonated beverages, beer, soy sauce and crackers, as a product of Maillard reaction. Thus, some soft drinks which use caramel coloring (class III and IV) will contain 4-MI [1]. An increased incidence of lung tumors was reported in mice, but not rats, exposed to levels of 4-MI in their diet that far exceed (more than 10,000 times) human dietary intake [2, 3]. International Agency for Research on Cancer (IARC) classified 4-MI as group 2B carcinogen based on the animal data [1, 4]. 4-MI does not induce mutation in *Salmonella* and does not induce micronuclei in rodent peripheral erythrocytes or bone marrow cells [2, 5]. Different evaluations have been made on the genotoxicity of 4-MI, i.e., sufficient evidence for drawing clear negative conclusion or not, among regulatory bodies. Clarification of factor of the difference is important for regulatory decisions with transparency and fairness in chemical management and control.

## 4-Methylimidazole (4-MI)

4-MI (CAS RN 822-36-6) is light yellow crystalline solid. It is used as a chemical intermediate, raw material or component in the manufacture of pharmaceuticals, photographic chemicals, dyes and pigments, agricultural chemicals and rubber. 4-MI is formed as a result of the interaction of ammonia with reducing sugars [1]. Therefore, it will be found as byproduct in some foods and beverages during the normal cooking process associated with heat and browning. 4-MI also forms as a trace impurity during the manufacturing of class III (ammonia caramel) and class IV (sulphite-ammonia caramel) caramel coloring [3]. Thus, 4-MI was identified in some types of soft drinks [6, 7].

## Carcinogenicity of 4-MI

In 2007, US National Toxicology Program (NTP) issued a report of results from toxicology and carcinogenesis studies of 4-MI in rats and mice [2]. A 2-year study in rats was inconclusive regarding carcinogenicity, but a 2-year mouse study showed an increased incidence of certain lung tumors (alveolar/bronchiolar neoplasms). Based on the NTP bioassay data and other relevant data, IARC assigned 4-MI as possibly carcinogenic to humans (group 2B) [1].

## Genotoxicity of 4-MI

Genotoxicity data on 4-MI is summarized in Table 1. 4-MI was negative in bacterial reverse mutation assay with *Salmonella typhimurium* TA97, TA98, TA100, TA1535, TA1537 or TA1538 up to 10,000 μg/plate in the presence or absence of S9 from rat or hamster liver, or *Escherichia coli* WP2 uvrA. 4-MI did not induce micronucleated erythrocytes in male or female mice treated by feeding up to 10,000 ppm (3200 or 1900 mg/kg/day, respectively) for 92 days in the peripheral blood micronucleus test. 4-MI was also evaluated in bone marrow micronucleus test in male rats or mice treated by intraperitoneal injection (three times once a day) up to 100 or 200 mg/kg, respectively. No increase in micronuclei was observed in the rats and mice (Table 1) [1, 5].

**Table 1:** Genotoxicity data on 4-methylimidazole

Test	System and conditions	Dose	Result	Reference
Bacterial reverse mutation test	*Salmonella typhimurium* TA97, TA98, TA100, TA1535, TA1537 and TA1538. With and without S9 from rat or hamster liver.	100 – 10,000 μg/plate	Negative	[1, 5]
Bacterial reverse mutation test	*Escherihia coli* WP2 uvrA. With and without S9.	9.77 – 5000 μg/plate	Negative	[5]
Bacterial reverse mutation test	*Salmonella typhimurium* TA98, TA100, TA1535, TA1537 and TA102. With and without S9 from rat liver or lung, or mouse liver or lung.	5 – 5000 μg/plate	Negative	[14]
SAR analysis	Three softwares including Osiris, ToxTree and DEREK.	Not applicable	Negative	[13]
Micronucreus assay	Male and female mouse peripheral blood. Diet for 92 days.	625 – 10,000 ppm (up to 3200 mg/kg/day for male, 1900 mg/kg/day for female)	Negative	[1, 5]
Micronucreus assay	Male mouse bone marrow. Three intraperitoneal injections.	25 – 200 mg/kg	Negative	[1, 5]
Micronucreus assay	Male rat bone marrow. Three intraperitoneal injections.	25 – 100 mg/kg	Negative	[1, 5]

*SAR* structure-activity relationship

## Regulatory perspectives of genotoxic concern of 4-MI

### NTP

It is unlikely that an alkylating intermediate is involved in mouse lung carcinogenesis in view of the genotoxicity study findings that 4-MI is not mutagenic in *S. typhimurium* and does not induce micronuclei in mouse peripheral blood erythrocytes or rat and mouse bone marrow cells. The mechanism of action of 4-MI in mouse lung tumorigenesis is not clear [8].

### IARC

4-MI induced neither mutations nor chromosomal aberrations in vitro or in vivo. The mechanism of action of 4-MI in mouse lung tumorigenesis is not clear [1].

### California Environmental Protection Agency (CEPA)

No Significant Risk Level (NSRL) for 4-MI has been calculated to be 29 μg/day under the regulation of Proposition 65. Proposition 65 (the Safe Drinking Water and Toxic Enforcement Act of 1986) is a California law which intends to protect California citizens and the State’s drinking water sources from chemicals known to cause cancer, birth defects or other reproductive harm, and to inform citizens about exposures to such chemicals [9]. The NSRL is defined as the daily intake level posing a 10^−5^ lifetime risk of cancer. Neither the mechanism(s) nor mode(s) of action of carcinogenicity of 4-MI is known. Though the available literature has provided little evidence for the genotoxicity of 4-MI, the testing has not been adequately comprehensive to rule out a genotoxic mode of action (MOA), particularly in the lung. Since the lung is a primary target tissue in mice, it may be more appropriate to use a metabolic activation system derived from pulmonary tissue. In addition, the implications of the negative results in bone marrow and peripheral blood erythrocytes are unclear. There is not sufficient evidence to justify departing from the default assumption. Thus, CEPA has adopted in its assessment that the carcinogenic effect of 4-MI is not a threshold mechanism [5].

### European Food Safety Authority (EFSA)

EFSA considered that the carcinogenic effect of 4-MI seen in mice in this study was thresholded, based on the lack of genotoxicity of 4-MI, also noting that alveolar/bronchiolar neoplasms occur spontaneously at high incidence in B6C3F1 mice. EFSA concluded therefore that the intermediate dose of 625 mg 4-MI/kg diet, equivalent to 80 mg 4-MI/kg bw/day could be considered to be a non-observed adverse effect level (NOAEL) in this study [10].

### US Food and Drug Administration (FDA)

NTP carcinogenicity studies were conducted in rodents at levels of 4-MI that far exceed current estimates of human exposure to 4-MI from the consumption of class III and class IV caramel coloring in food products such as colas. In 2012, EFSA re-evaluated the consumer exposure to 4-MI from the use of caramel colors, and reaffirmed its 2011 conclusion [11]. EFSA also noted that 4-MI does not appear to cause DNA mutations (genotoxicity) and that the type of tumors observed in the mice from the NTP study can occur spontaneously in these animals. For these reasons, EFSA concluded that they had no concerns about Europeans being exposed to 4-MI from the use of caramel coloring in food [2, 10].

### German Federal Institute for Risk Assessment (BfR)

BfR checked the data on 4-MI and the opinion of EFSA. EFSA stated in its opinion that the carcinogenic effect of 4-MI in mice a threshold value can be accepted since it with 4-MI no genotoxic effects were observed. EFSA also pointed out that with 4-MI tumors observed in mice a comparatively high spontaneous rate. BfR agreed with EFSA’s opinion that the maximum quantities for 4-MI in caramel class III or IV according to the current state of science are completely harmless [12].

## Different regulatory perspectives and new genotoxicity data on 4-MI

The issue underlying regulatory perspectives of health concern of 4-MI is evaluation of genotoxic MOA in the mouse carcinogenicity of 4-MI. Though the mechanism of action of 4-MI in mouse lung tumorigenesis is not clear, regulatory bodies except for CEPA considered the carcinogenicity is not genotoxic MOA based on the existing data [3, 10, 11]. On the other hand, CEPA could not rule out possible genotoxic MOA in the target organ (i.e., lung); therefore, CEPA employed non-threshold mechanism on 4-MI carcinogenicity as default assumption [5]. Recently, new genotoxicity data on 4-MI has been published (Table 1) [13, 14]. Analysis with three structure-activity relationship software including Osiris, ToxTree and DEREK revealed that 4-MI is not genotoxic and carcinogenic [13]. In addition, 4-MI did not increase revertant colonies at doses tested up to 5000 μg/plate in *Salmonella typhimurium* strains TA98, TA100, TA1535, TA1537 and TA102 both in the absence and presence of exogenous metabolism, regardless of whether metabolic activity was provided by S9 from induced rat liver or lung or mouse liver or lung [14]. These new data and other existing genotoxicity data indicate that 4-MI is a non-genotoxic substance and the mechanism of induction of lung tumors in mice treated by 4-MI is highly unlikely due to genotoxicity. Therefore, application of the threshold mechanism to the 4-MI carcinogenicity is reasonable.

## Risk evaluation of 4-MI in caramel colors, foods or beverages

**Fig. 1:**
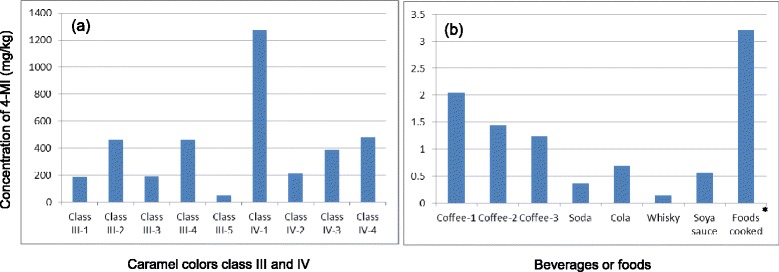
Concentration of 4-methylimidazole in caramel colors class III and IV (**a**), or in some beverages or foods (**b**). Each caramel color or beverage/food which contains maximum level of 4-MI was selected among several samples. Class III-1 to III-5 and IV-1 to IV-4 mean different samples among class III or IV caramel color products. Data were modified from IARC Monographs 101 [1]. *: Foods cooked in soya sauce

As 4-MI is not genotoxic, estimated tolerable daily intake will be calculated 0.8 mg/kg/day (48 mg in terms of 60 kg body weight per person), based on the NOAEL of 80 mg/kg/day evaluated by EFSA (safety factor 100) [10]. When 500 mL of a certain type of soft drink which contains 0.7 mg 4-MI/kg will be drunken, a maximum intake of 4-MI will be 0.35 mg/person (Fig. 1b); this exposure level does not cause any risk of 4-MI contained in beverages which use caramel colors class III or IV. Low level of exposure of 4-MI is the reason that EFSA states no concerns of health effect by 4-MI derived from caramel colors. EFSA considered it would be prudent to reduce 4-MI level as much as technology feasible [10]. Therefore, the maximum level of the constituent 4-MI, found in caramel class III and class IV only, was restricted to <250 mg/kg caramel on a color intensity basis in the year 2012 [15]. The specification was defined as much as technologically feasible (Fig. 1a) [15]. Exposure level of 4-MI from soft drink (e.g., cola) is not high due to low concentration and small amount of cola intake. The mechanism by which 4-MI induces lung tumors in mice but not rats is unknown. The hypothesis that 4-MI and styrene induce lung tumors by the same MOA (i.e., CYP2F2 metabolic activation) has not been supported by recent investigations [16]. Further investigation on the mechanism of the mouse carcinogenicity will help the risk assessment of 4-MI.

## Conclusion

Evaluation of involvement of genotoxic mechanism in chemical carcinogenesis is important for the regulatory decisions in the chemical control and management. Different approach will be adopted based on the evaluation, i.e., threshold or non-threshold approach. Recent genotoxicity data supports that 4-MI is non-genotoxic carcinogen. Therefore, threshold level can be applied. There is no health concern on 4-MI from soft drinks which use certain types of caramel colors. Risk evaluation of 4-MI from other foods which contain higher level of 4-MI might be needed.

## Abbreviations

4-MI, 4-Methylimidazole; BfR, German Federal Institute for Risk Assessment; CEPA, US California Environmental Protection Agency; EFSA, European Food Safety Authority; FDA, US Food and Drug Administration; IARC, International Agency for Research on Cancer; MOA, Mode of Action; NOAEL, Non-observed adverse effect level; NSRL, No significant risk level; NTP, US National Toxicology Program
